# Supplementary material for: Decryption of sequence, structure, and functional features of SINE repeat elements in SINEUP non-coding RNA-mediated post-transcriptional gene regulation
Source: Nat Commun. 2024 Feb 21;15:1400. doi: 10.1038/s41467-024-45517-3 (PMC10881587; doi:10.1038/s41467-024-45517-3)
Supplement: Supplementary file 1 — Supplementary Information [file 41467_2024_45517_MOESM1_ESM.pdf]

Decryption of sequence, structure, and functional features of SINE repeat elements in SINEUP non-coding RNA–mediated post-transcriptional gene regulation

Harshita Sharma[1], Matthew N Z Valentine[1], Naoko Toki[1], Hiromi Sueki Nishiyori[1], Stefano Gustincich[2], Hazuki Takahashi[1]* and Piero Carninci[1,3]*

1 Laboratory for Transcriptome Technology, RIKEN Center for Integrative Medical Sciences, Yokohama, Kanagawa, 230-0045, Japan
2 Department of Neuroscience and Brain Technologies, Istituto Italiano di Tecnologia, Genova, Italy
3 Human Technopole, Milan, 20157, Italy
* Corresponding authors
E-mails: carninci@riken.jp; piero.carninci@fht.org; hazuki.takahashi@riken.jp

Supplementary Information-

Supplementary Fig. 1

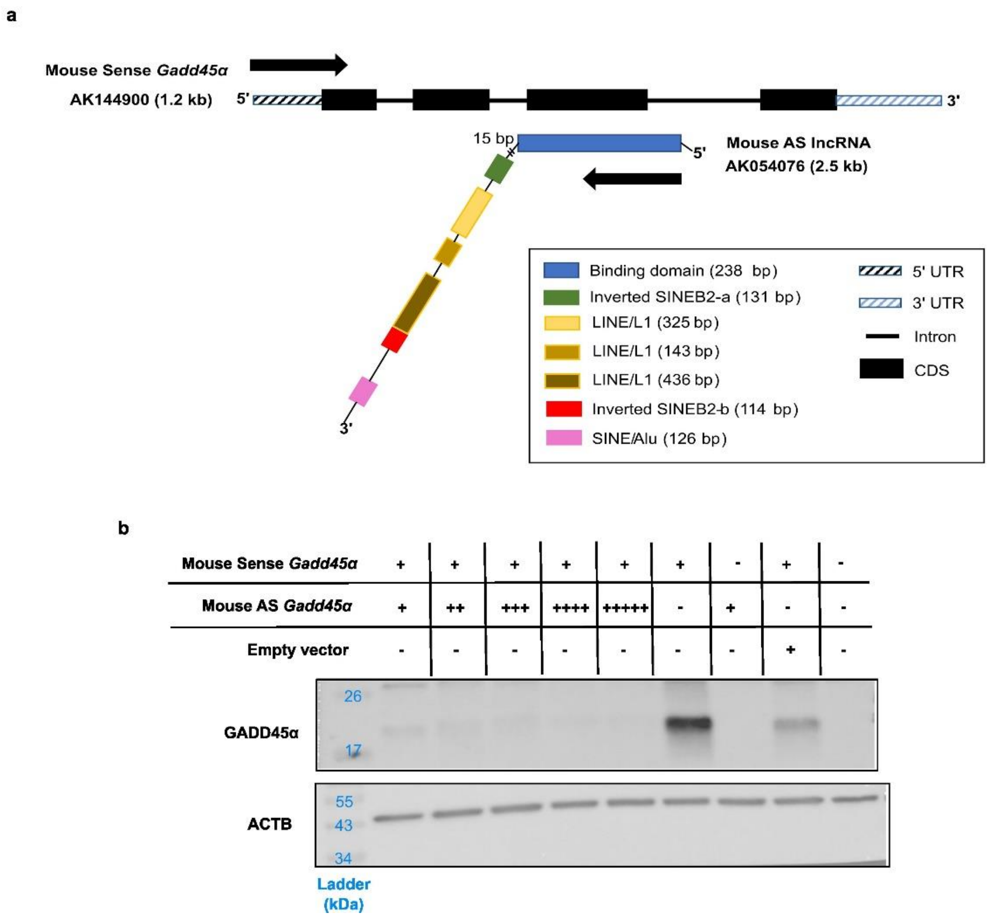

Supplementary Fig. 1. Effect of mouse natural antisense *Gadd45α* lncRNA on sense *Gadd45α* protein expression. a Molecular features of mouse sense/antisense *Gadd45α* RNAs. Antisense (AS) *Gadd45α* contains a sequence complementary to intron 2, exon 3, and intron 3 of sense Gadd45α mRNA,two

inverted SINEB2s, three LINE, and one SINE-Alu elements. **b** Western blot analysis of sense Gadd45α protein expression in HEK293T/17 cells co-transfected with sense/antisense *Gadd45α* for 24 h. Empty vector, negative control; plasmid transfection at + 0.7 pmol; ++ 0.8 pmol; +++ 0.9 pmol; ++++ 1.0 pmol; - not transfected. ACTB is the loading control. N = 1 biologically independent experiment. Source data are provided as a Source Data file.

Supplementary Fig. 2

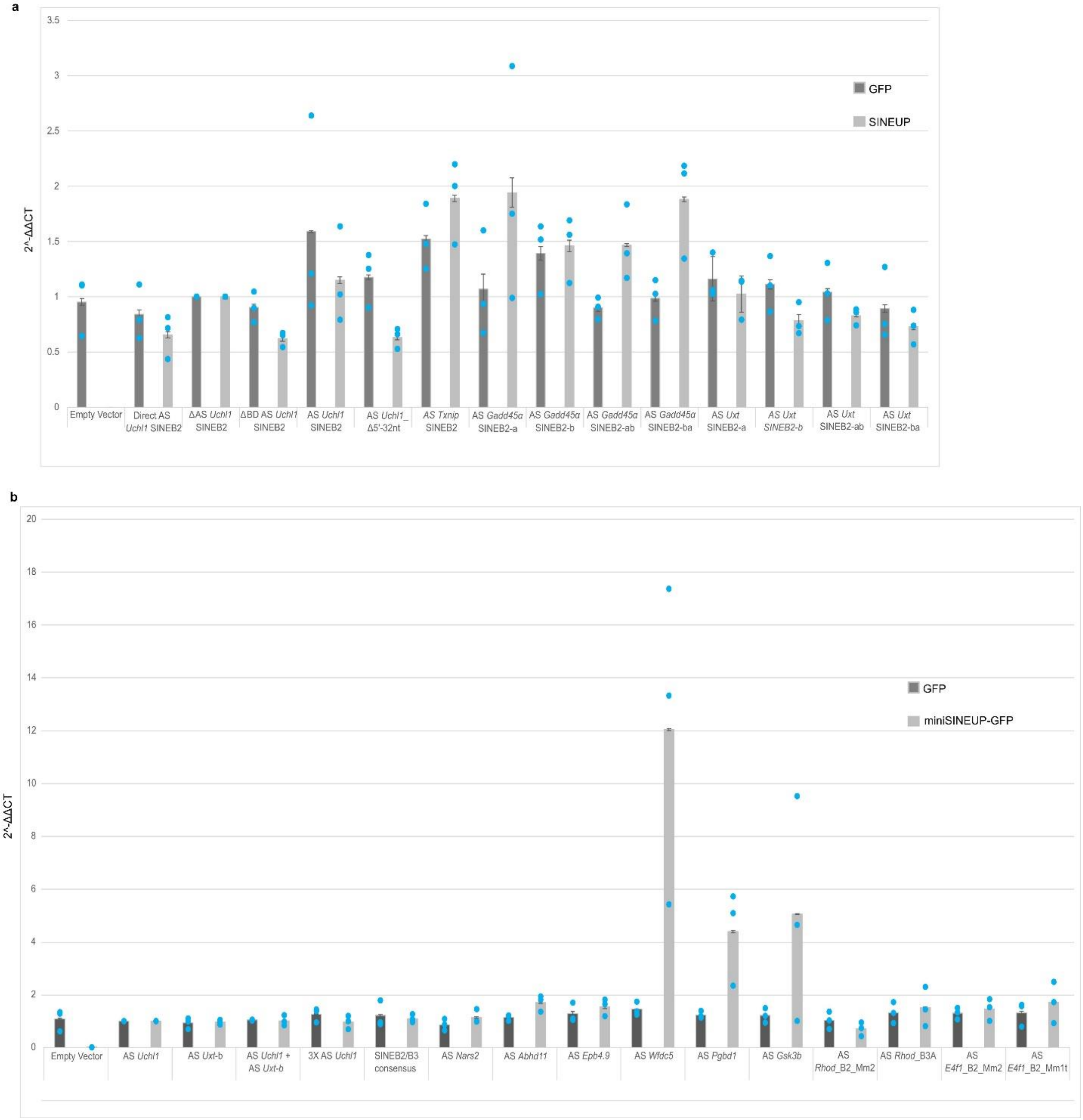

Supplementary Fig. 2. qRT-PCR results for GFP mRNA (black bars) and SINEUP-GFP RNA (grey bars) expression. Expression values are normalized to human GAPDH mRNA. Data were analyzed by using the $\Delta\Delta C_T$ method. RNA was extracted from (**a**) long SINEUPs (see description of terms in Figure 1c), with ΔAS *Uchl1* SINEB2 = 1 and from (**b**) MiniSINEUP-GFP (described in Figure 2b), with AS *Uchl1* = 1. Data are mean +/- SEM for three biologically independent experiments. Data points for independent experiments are represented by blue dots. AS, antisense. Source data are provided as a Source Data file.

Supplementary Fig. 3

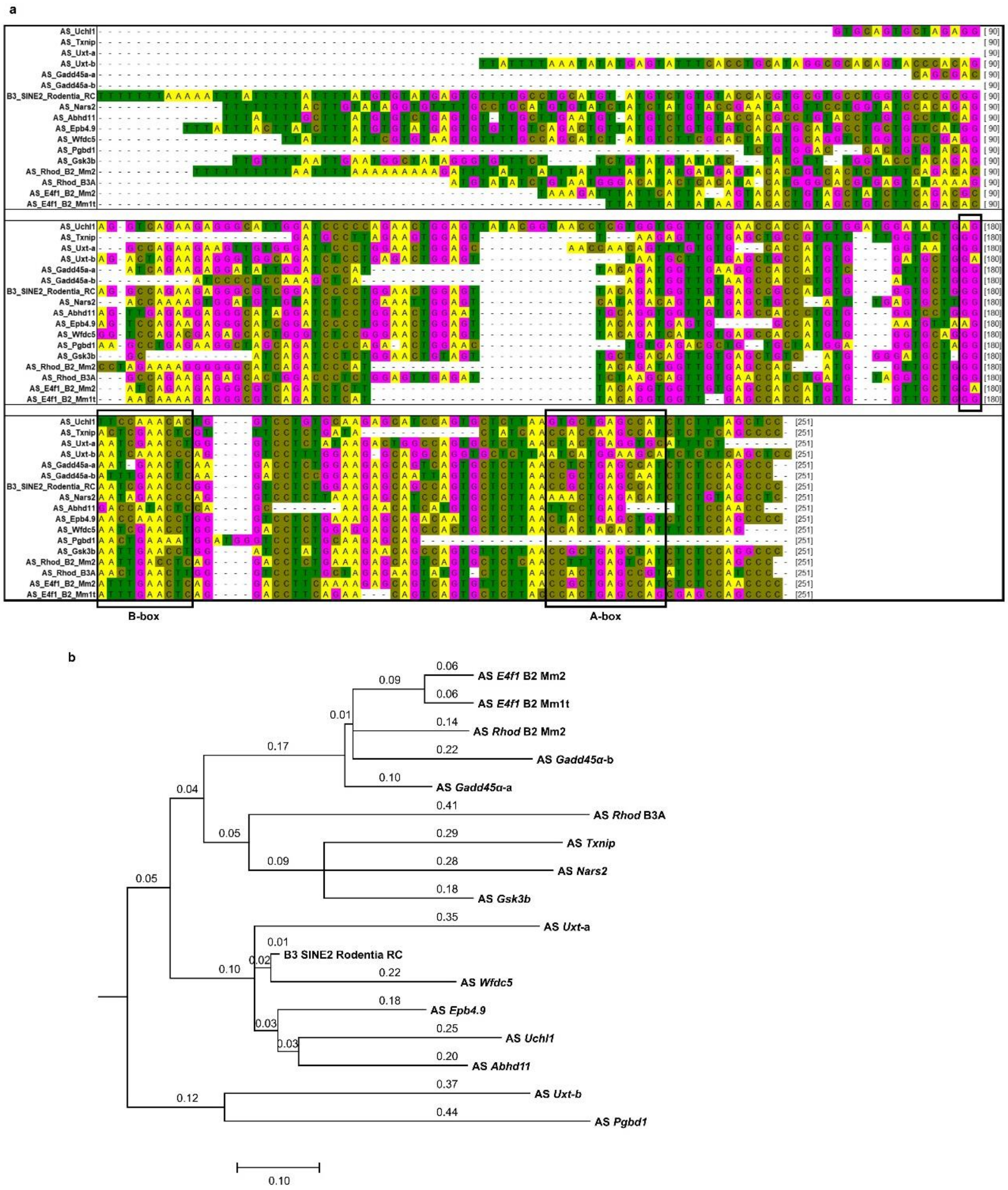

Supplementary Fig. 3. Multiple sequence alignment and phylogenetic analysis of SINEB2 RNA sequences. **a** ClustalW sequence alignment of mouse SINEB2 RNA sequences used as effector domain in long and mini SINEUPs. Gaps in the alignment are indicated by -. Bases are highlighted in four different colors. Black squares denote A and B boxes. Alignment shows the sequence variation within members of the same family of SINE. **b** SINEB2 sequence alignment–based molecular phylogenetic analysis by the Maximum Likelihood method (Tamura 3-parameter model) to infer the evolutionary history. The tree with the highest log likelihood (−2415.7806) is shown. The tree is drawn to scale, with branch lengths measured

in the number of substitutions per site. AS, antisense; B3 SINE2 Rodentia RC, the consensus sequence for SINEB2 subfamily B3 from the Repbase database.

Supplementary Fig. 4

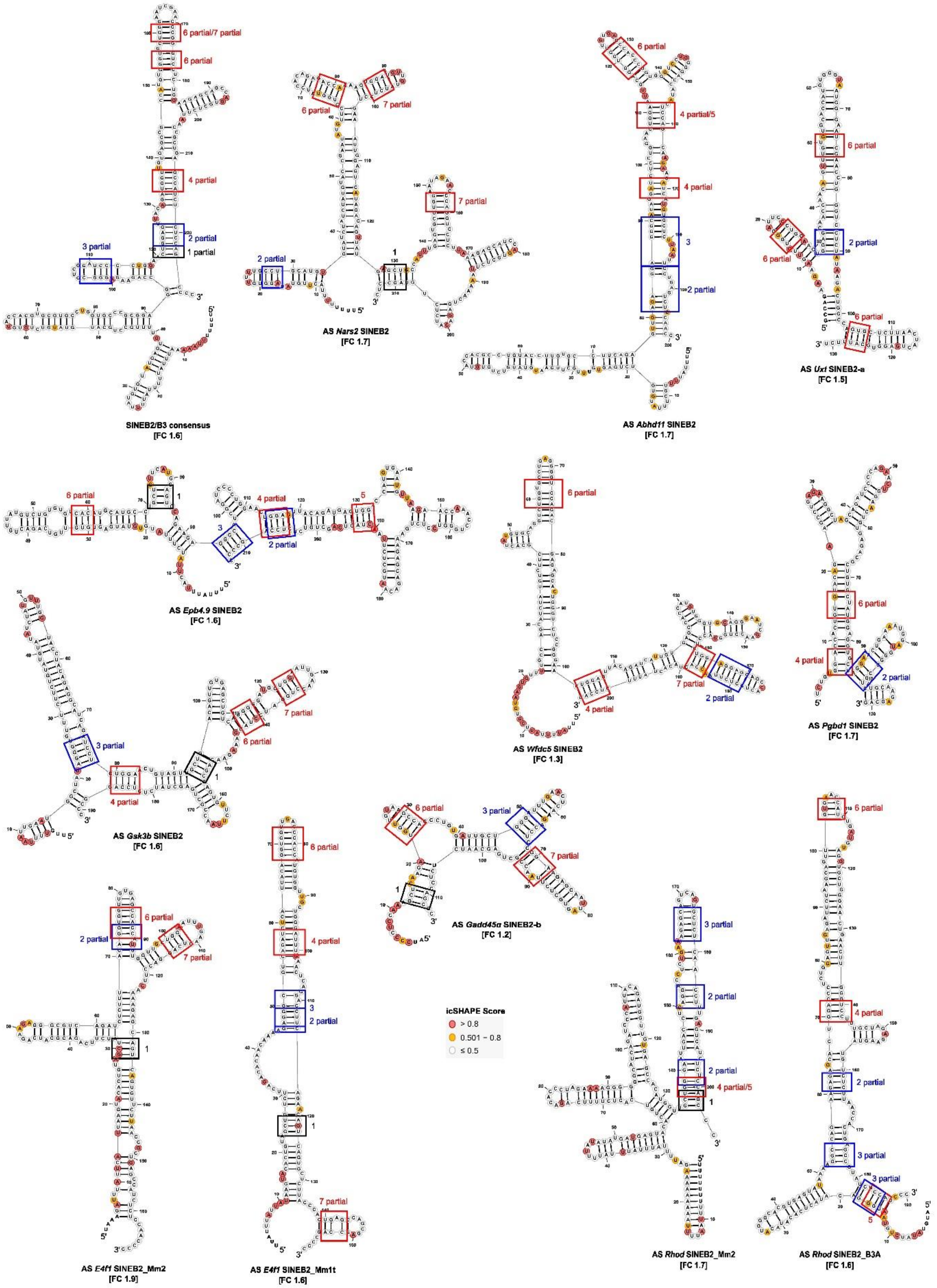

Supplementary Fig. 4. icSHAPE 2D structure models of mouse SINEB2 RNAs from natural antisense transcripts that are tested as effector domain in SINEUPs. Regions that match the SINEUP motifs are marked by squares with their corresponding motif number. Blue square, AGG type motif; red square, UGG type motif. FC, GFP protein fold-change induced by the respective SINEB2. Nucleotide color indicates normalized icSHAPE reactivity score: red, ≥ 0.8; yellow, from above 0.5 to 0.8; white encircled with grey, ≤ 0.5; bases not encircled, no icSHAPE data available. AS, antisense.

Supplementary Fig. 5

a

S. No.	SINEUP Motif	CoSSMos motif match	PDB ID	Positions	Molecule
1.	Motif 6	GGUGG<br>UCCC	5A2Q	G1324-G1325-U1326-G1327-G1328<br>U1504-C1503-C1502-C1501	18S rRNA
2.	Motif 2	GGAGG<br>CCCC	5JB3	G999-G1000-A1001-G1002-G1003<br>C1170-C1169-C1168-C1167	16S rRNA

b

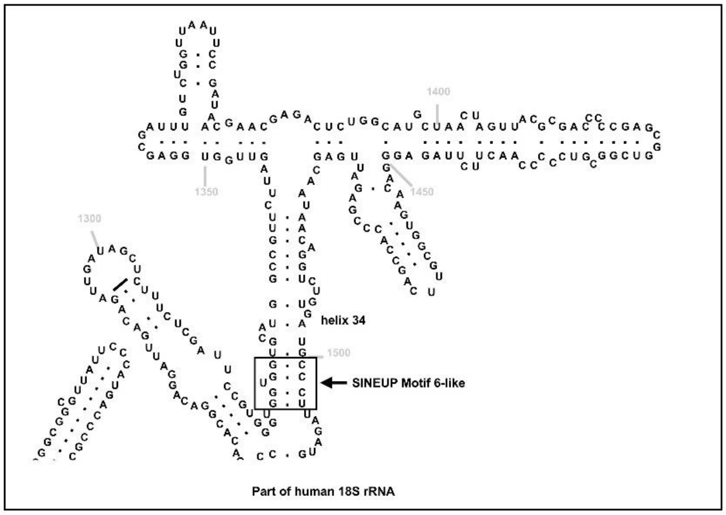

Supplementary Fig. 5. Similarity of SINEUP motifs to rRNA 3D structure motifs. a Hits found in the Characterization of Secondary Structure Motifs database for 3D motifs that match the SINEUP structure motifs. **b** The region similar to SINEUP motif 6 in helix 34 of human 18S rRNA is framed in black and marked with an arrow.

Supplementary Fig. 6

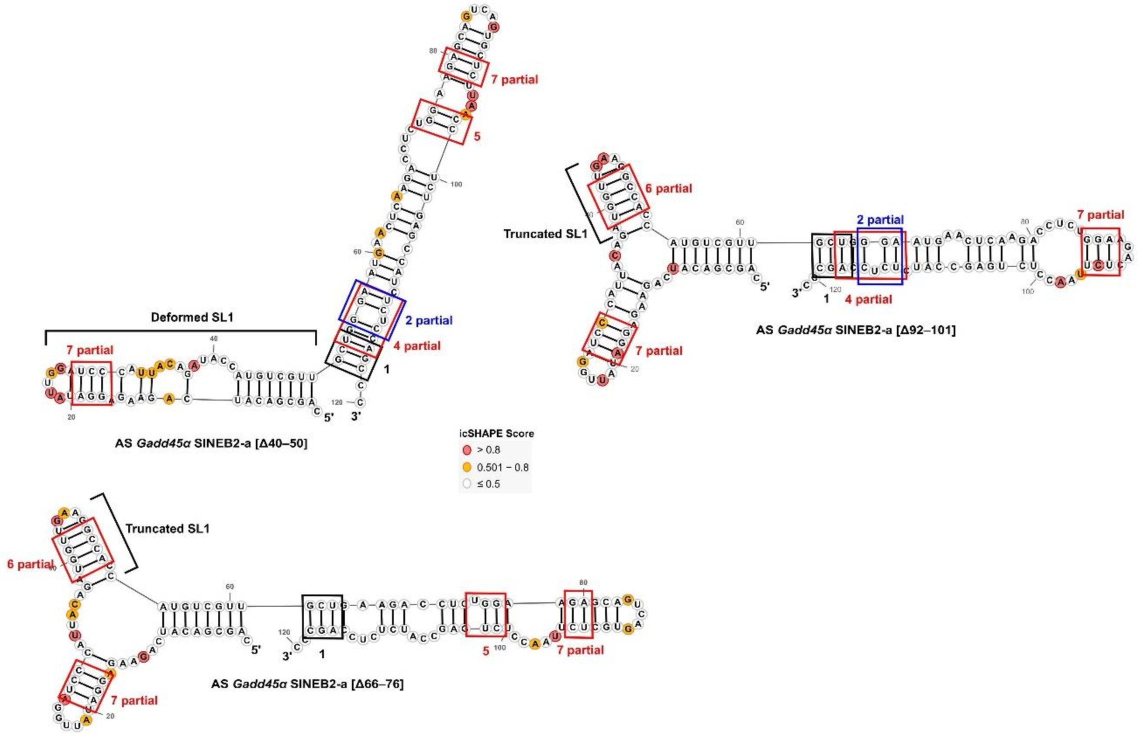

Supplementary Fig. 6. SINEUP structure motifs in functionally inactive antisense (AS) *Gadd45α* SINEB2-a deletion mutants. icSHAPE derived structures of AS *Gadd45α* SINEB2-a deletion mutants [Δ40–50], [Δ66–76], and [Δ92–101]. Regions that match conserved SINEUP structure motifs are marked by different colors and numbers. Δ = deletion. Nucleotide color indicates normalized icSHAPE reactivity score: red, ≥ 0.8; yellow, from above 0.5 to 0.8; white encircled with grey, ≤ 0.5; bases not encircled, no icSHAPE data available. AS, antisense.

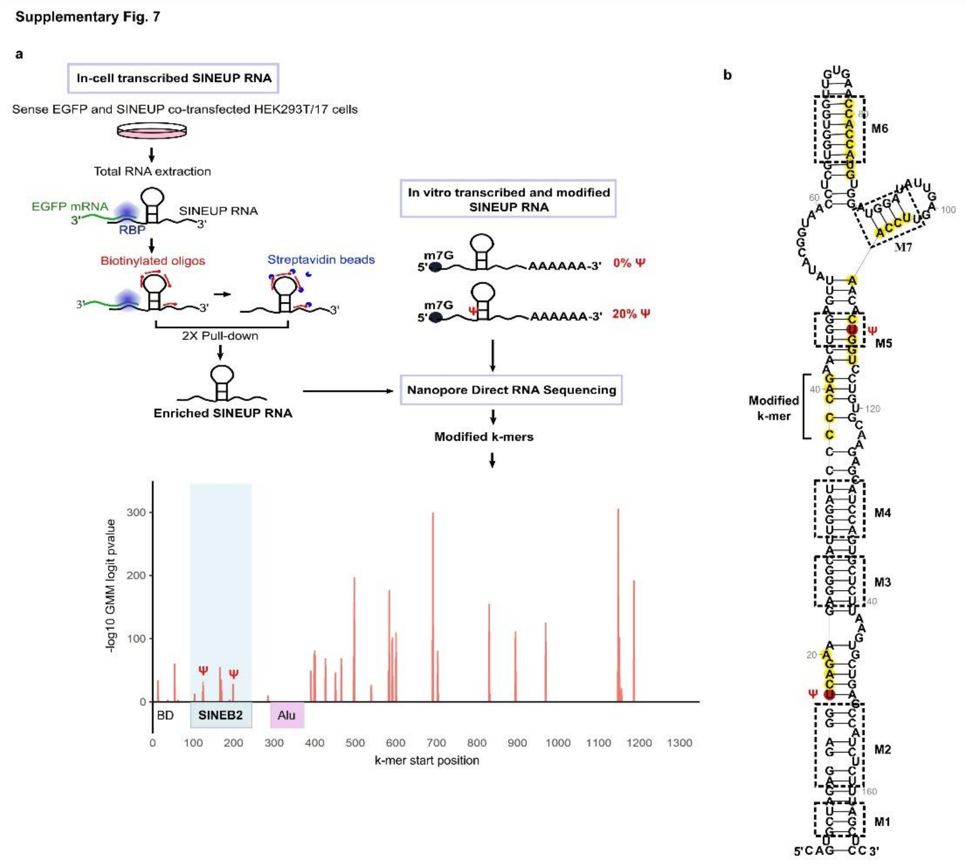

Supplementary Fig. 7. Discovery of modified bases in SINEB2 RNA. a An overview of SINEUP RNA modification analysis. SINEUP-GFP RNA (containing antisense Uchl1 SINEB2) transcribed in vitro was modified with 20% pseudouridine (Ψ), and a non-modified (0% Ψ) sample was used as control. In parallel, SINEUP-GFP and sense EGFP plasmids were co-transfected in HEK293T/17 cells, then SINEUP RNA that was transcribed in-cell was captured and enriched using specific biotinylated oligo probes and pull-down (twice) on streptavidin-coated magnetic beads. Purified samples of SINEUP-GFP RNA transcribed in-cell or transcribed and modified in vitro were sequenced by Nanopore direct RNA sequencing. Profiles of non-modified (0% Ψ) and modified SINEUP-GFP RNA transcribed in vitro were compared with that transcribed in-cell, and k-mers corresponding to the modified positions were identified in the in-cell transcripts. The red peaks in the graph mark the position of modified k-mers on the SINEUP transcript. The SINEB2 region is shaded in blue, and verified Ψ sites are marked. BD, binding domain. **b** Modified k-mers overlaid on 2D structure of antisense *Uchl1* SINEB2. k-mers are highlighted in yellow, Ψ sites are in red. SINEUP motifs are marked in black dashed squares with motifs 1–7 labelled as M1–M7.

Supplementary Fig. 8

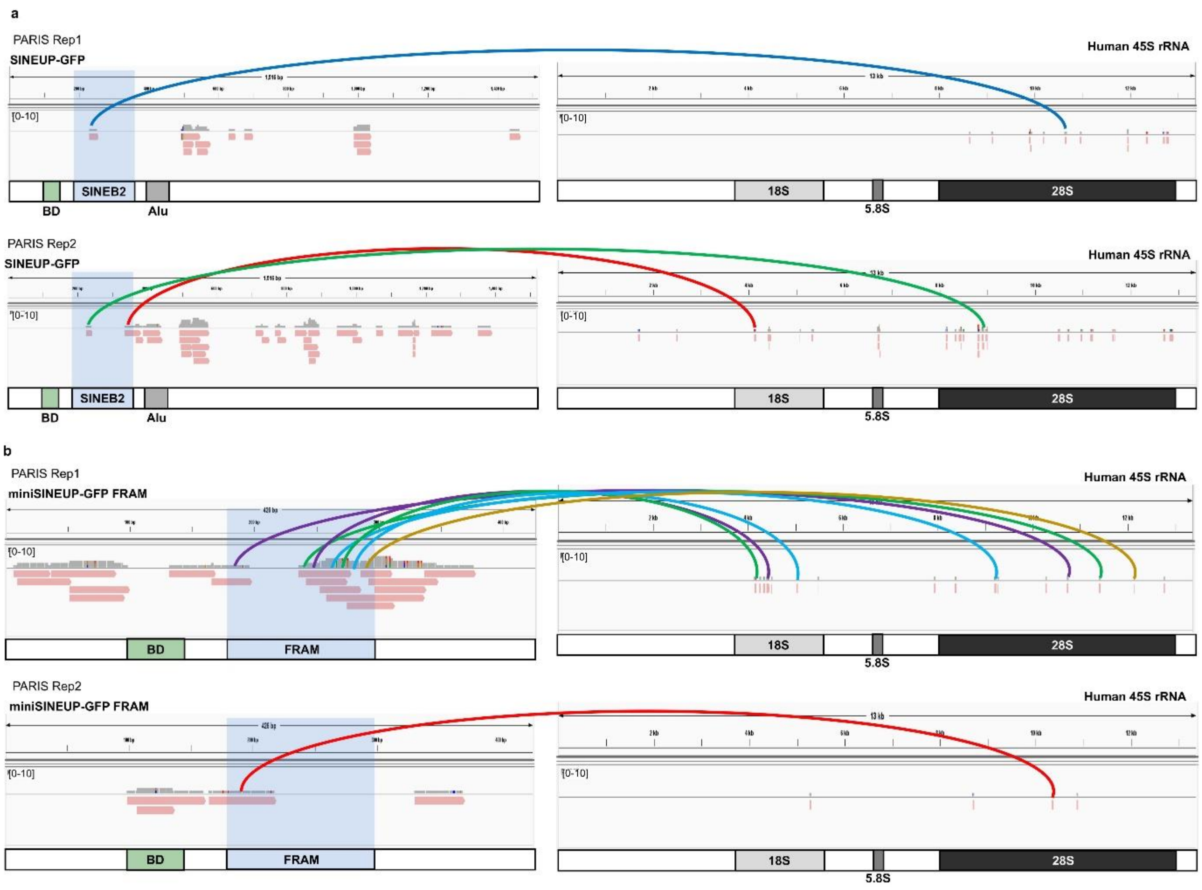

Supplementary Fig. 8. SINEUP-rRNA duplex reads in PARIS sequencing data. a SINEUP-GFP (of antisense *Uchl1* SINEB2) RNA duplex reads with 18S, 5.8S, and 28S rRNA. Two biological replicates of the PARIS experiment are shown as separate tracks. SINEB2-rRNA interactions shown in Fig. 4 are indicated by respective colored arcs. **b** miniSINEUP-GFP FRAM RNA-rRNA duplex reads. PARIS rep1 and rep 2 are two biological replicates. The FRAM RNA-rRNA interactions displayed in Fig. 5 are marked by arcs in their respective colors.

Supplementary Fig. 9

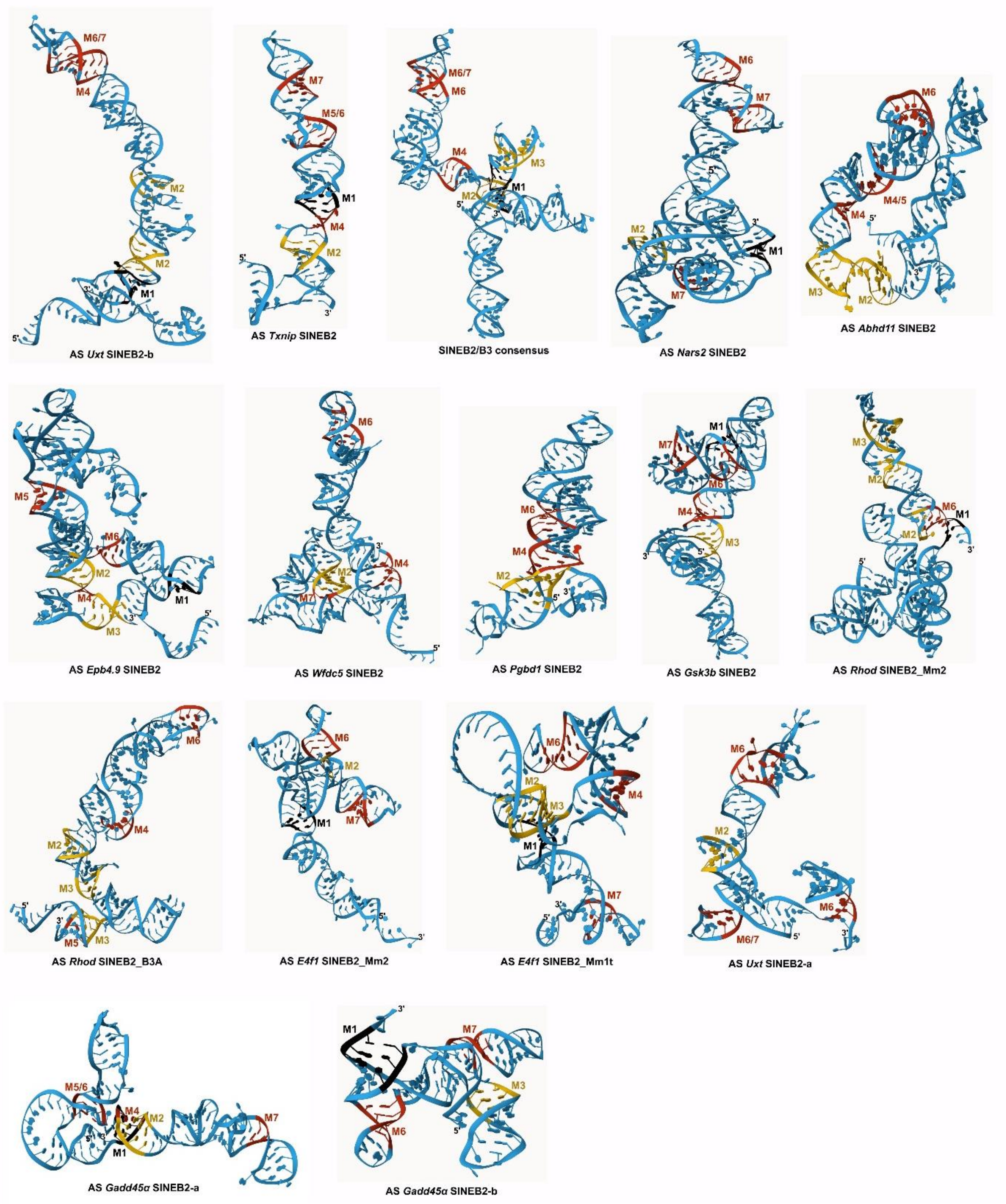

Supplementary Fig. 9. SINE RNA predicted 3D structure models based on in-cell 2D structures of mouse SINEB2 RNAs. SINEUP structure motifs are denoted from M1 to M7. AS: antisense.

Supplementary Fig. 10

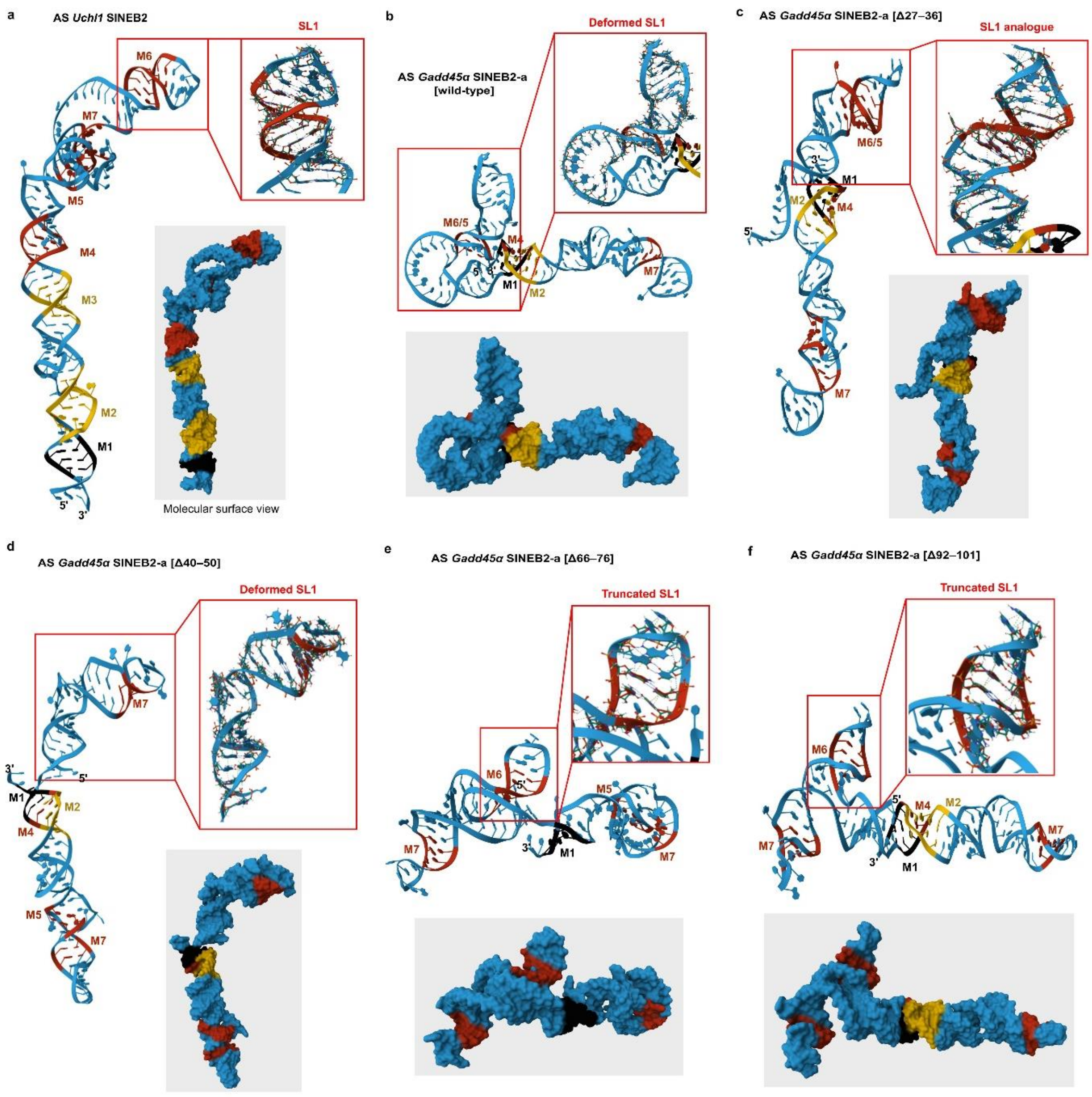

Supplementary Fig. 10. Comparison of icSHAPE data-driven predicted 3D structure models of wild-type AS *Gadd45α* SINEB2-a and its deletion mutants with functional AS *Uchl1* SINEB2. a AS *Uchl1* SINEB2 3D model predicted from whole cell icSHAPE data. Predicted 3D models with enlarged view of the corresponding SL1 regions for AS *Gadd45α* SINEB2-a **b** wild-type (functionally inactive), its (**c**) functionally active deletion mutant [Δ27–36], and non-functional mutants (d) [Δ40–50], (e) [Δ66–76], and (f) [Δ92–101]. SINEUP structure motifs M1–M7 are marked. Red frame shows zoomed-in view of the region corresponding to SL1. The molecular surface views of the 3D models are shaded in gray. Δ = deletion.
