# Supplementary material for: Decryption of sequence, structure, and functional features of SINE repeat elements in SINEUP non-coding RNA-mediated post-transcriptional gene regulation
Source: Nat Commun. 2024 Feb 21;15:1400. doi: 10.1038/s41467-024-45517-3 (PMC10881587; doi:10.1038/s41467-024-45517-3)
Supplement: Supplementary file 3 — Inventory of Supporting Information [file 41467_2024_45517_MOESM3_ESM.pdf]

Inventory of Supplementary Information-

Supplementary Data 1: SINEB2 sequences tested as effector domain (ED) in (**a**) long SINEUP and (**b**) in miniSINEUP-GFP.

Supplementary Data 2: List of base-stacking positions in SINE RNA 3D models.

Supplementary Data 3: List of non-canonical interactions identified in SINE RNA 3D models.

Supplementary Data 4: Intermolecular interactions of SINEUPs with RNAs other than rRNA in PARIS2 data.

Supplementary Data 5: **a** Primers used for QuikChange II site-directed mutagenesis. **b** Primers used for PCR-cloning of miniSINEUP-GFP constructs.

Supplementary Data 6: Oligos used in (**a**) icSHAPE, (**b**) seCLIP, and (**c**) PARIS library preparation.
