# Supplementary material for: Decryption of sequence, structure, and functional features of SINE repeat elements in SINEUP non-coding RNA-mediated post-transcriptional gene regulation
Source: Nat Commun. 2024 Feb 21;15:1400. doi: 10.1038/s41467-024-45517-3 (PMC10881587; doi:10.1038/s41467-024-45517-3)
Supplement: Supplementary file 10 — Reporting Summary [file 41467_2024_45517_MOESM10_ESM.pdf]

nature portfolio

Corresponding author(s): Hazuki Takahashi and Piero Carninci

Last updated by author(s): Jan 6, 2024

Reporting Summary

Nature Portfolio wishes to improve the reproducibility of the work that we publish. This form provides structure for consistency and transparency in reporting. For further information on Nature Portfolio policies, see our Editorial Policies and the Editorial Policy Checklist.

Statistics

For all statistical analyses, confirm that the following items are present in the figure legend, table legend, main text, or Methods section.

n/a	Confirmed	
☐	☒	The exact sample size (*n*) for each experimental group/condition, given as a discrete number and unit of measurement
☐	☒	A statement on whether measurements were taken from distinct samples or whether the same sample was measured repeatedly
☐	☒	The statistical test(s) used AND whether they are one- or two-sided<br>*Only common tests should be described solely by name; describe more complex techniques in the Methods section.*
☐	☒	A description of all covariates tested
☐	☒	A description of any assumptions or corrections, such as tests of normality and adjustment for multiple comparisons
☐	☒	A full description of the statistical parameters including central tendency (e.g. means) or other basic estimates (e.g. regression coefficient) AND variation (e.g. standard deviation) or associated estimates of uncertainty (e.g. confidence intervals)
☐	☒	For null hypothesis testing, the test statistic (e.g. *F*, *t*, *r*) with confidence intervals, effect sizes, degrees of freedom and *P* value noted<br>*Give P values as exact values whenever suitable.*
☒	☐	For Bayesian analysis, information on the choice of priors and Markov chain Monte Carlo settings
☒	☐	For hierarchical and complex designs, identification of the appropriate level for tests and full reporting of outcomes
☒	☐	Estimates of effect sizes (e.g. Cohen's *d*, Pearson's *r*), indicating how they were calculated

Our web collection on statistics for biologists contains articles on many of the points above.

Software and code

Policy information about availability of computer code

Data collection	uman 18S rRNA structure (ID: URS0000SA14E2) and 28S rRNA structure (ID: URS0000C873C2) were taken from the RNAcentral database (https:/ /rnacentral.org/).<br>hg38 masked genome used to check SINEUP-rRNA chimera- https://drive.google.com/drive/folders/lwHSC-mfljNNCIXrVqMugqVmDVT4Crxzz
Data analysis	Western Band intensities were measured by using- Image J version 1.48 software (National Institutes of Health)<br>qPCR data was analyzed by- StepOne v2.3 and SDS v2.4 software<br>Phylogenetic analysis was performed by- MEGA version 5 software<br>3D RNA structure prediction by- RNAComposer (https://rnacomposer.cs.put.poznan.pl/)<br>3D RNA structure models visualization by- Mai* tool of RCSB PDB (https://www.rcsb.org/)<br>3D structure models analyzed by- RNApdbee 2.0 (http://rnapdbee.cs.put.poznan.pl/)<br>Mapped reads were analyzed by- integrated genome viewer (IGV v2.11.1)<br>icSHAPE pipeline- https://github.com/qczhang/icSHAPE<br>ENCODE eCLIP project pipeline- https://www.encodeproject.org/eclip/<br>clipper v0.2.0- https://github.com/Yeolab/clipper<br>merge_peaks github repository- https://github.com/Yeolab/merge_peaks<br>Nanocompore pipeline- https://github.com/tleonardi/nanocompore/<br>Peak-ea lii ng algorithm- https://gist.gith ub.com/tleonardi/0bb3 le6a380e5766f04f 4e 197 d36b38e<br>PARIS2 pipeline- https://github.com/minjiezhang-usc/PARIS2

For manuscripts utilizing custom algorithms or software that are central to the research but not yet described in published literature, software must be made available to editors and reviewers. We strongly encourage code deposition in a community repository (e.g. GitHub). See the Nature Portfolio guidelines for submitting code & software for further information.

Data

Policy information about availability of data

All manuscripts must include a data availability statement. This statement should provide the following information, where applicable:

- Accession codes, unique identifiers, or web links for publicly available datasets
- A description of any restrictions on data availability
- For clinical datasets or third party data, please ensure that the statement adheres to our policy

The icSHAPE sequencing data were deposited and available in Gene Expression Omnibus (GEO) under accession numbers GSE146407 [https://www.ncbi.nlm.nih.gov/geo/query/acc.cgi?acc=GSE146407] (for whole cell icSHAPE libraries of SINEUP-GFP) and GSE224534 [https://www.ncbi.nlm.nih.gov/geo/query/acc.cgi?acc=GSE224534] (for whole cell icSHAPE libraries of miniSINEUP-GFP and nuclear-cytoplasmic fractionated icSHAPE libraries), and GSE243220 [https://www.ncbi.nlm.nih.gov/geo/query/acc.cgi?acc=GSE243220] (for whole cell icSHAPE libraries of antisense Gadd45α SINEB2-a deletion mutants). The PARIS2 sequencing data and SINEUP RNA modification data are accessible through GEO accession numbers GSE224533 [https://www.ncbi.nlm.nih.gov/geo/query/acc.cgi?acc=GSE224533] and GSE224018 [https://www.ncbi.nlm.nih.gov/geo/query/acc.cgi?acc=GSE224018] respectively. Nuclear-cytoplasmic fractionated seCLIP sequencing data is available through the GEO accession number GSE227250 [https://www.ncbi.nlm.nih.gov/geo/query/acc.cgi?acc=GSE227250]. Source data are provided with this paper.

Research involving human participants, their data, or biological material

Policy information about studies with human participants or human data. See also policy information about sex, gender (identity/presentation), and sexual orientation and race, ethnicity and racism.

Reporting on sex and gender	All of our experiments were performed on human embryonic kidney cell line (HEK293T/17, Cat# CRL11268, ATCC) that origination is female fetus.
Reporting on race, ethnicity, or other socially relevant groupings	We did not use human participants or human data. Therefore, this information is not applicable to our study.
Population characteristics	We did not use human participants or human data. Therefore, this information is not applicable to our study.
Recruitment	We did not use human participants or human data. Therefore, this information is not applicable to our study.
Ethics oversight	We did not use human participants or human data. Therefore, this information is not applicable to our study.

Note that full information on the approval of the study protocol must also be provided in the manuscript.

Field-specific reporting

Please select the one below that is the best fit for your research. If you are not sure, read the appropriate sections before making your selection.

☒ Life sciences ☐ Behavioural & social sciences ☐ Ecological, evolutionary & environmental sciences

For a reference copy of the document with all sections, see nature.com/documents/nr-reporting-summary-flat.pdf

Life sciences study design

All studies must disclose on these points even when the disclosure is negative.

Sample size	We selected number of SINEs-derived RNAs randomly. No prior statistical analysis was done to determine the sample size. Since this study focuses on comparative analysis of sequence, structure, and functional features of various SINE-derived RNAs, the current sample size is sufficient.
Data exclusions	We did not exclude any data.
Replication	All Western blot data was verified by 3 to 5 biological replicates. All the qPCR data was verified by 3 biological replicates. Two biological replicates each for icSHAPE, PARIS, and seCLIP libraries were prepared. In case of icSHAPE and seCLIP 2 biological replicates were merged for the analysis. The chimeric reads in the PARIS2 lacked reproducibility likely due to transient nature of such chimeric interactions in the cell and partly due to technical limitation of PARIS2 method.
Randomization	We used randomization in experimental design. For instance, we set control samples for all experiments and performed 3-5 times in different experimental setting as for biological replicates and technical replicates. All experiments were independently performed to ensure the randomization in experimental design.
Blinding	We used blinding procedures in experimental design. For instance, all western blotting analysis and RNA-seq library preparations were performed at least two people with independent procedures in this manuscript. The RNA sequencing dataset was generated in our institutional sequencing facility with blinding procedures. All analysis software has been already publicly available after blinding reviewing procedures and globally used in scientific communities.

Reporting for specific materials, systems and methods

We require information from authors about some types of materials, experimental systems and methods used in many studies. Here, indicate whether each material, system or method listed is relevant to your study. If you are not sure if a list item applies to your research, read the appropriate section before selecting a response.

Materials & experimental systems

n/a	Involved in the study	
☐	☒	Antibodies
☐	☒	Eukaryotic cell lines
☒	☐	Palaeontology and archaeology
☒	☐	Animals and other organisms
☒	☐	Clinical data
☒	☐	Dual use research of concern
☒	☐	Plants

Methods

n/a	Involved in the study	
☒	☐	ChIP-seq
☒	☐	Flow cytometry
☒	☐	MRI-based neuroimaging

Antibodies

Antibodies used	EGFP was detected by anti-GFP rabbit serum (Life Technologies, Cat# A-6455, Lot# 1495850) in 1:1000 dilution<br>ACTINB was detected by monoclonal anti-β-actin antibody (Sigma Aldrich, Cat#A5441, Batch# 014M4759) in 1:1000 dilution<br>Polyclonal Goat Anti-Rabbit (Dako Denmark A/S, Cat# P0448, Lot# 00065567) and Polyclonal Goat Anti-mouse (Dako Denmark A/S, Cat# P0447, Lot# 00095437) were used as secondary antibodies in 1:1000 dilution.<br>HNRNPK was detected by anti-hnRNPK antibody (Abcam, Cat# ab39975).
Validation	Validation data from manufacture is available at<br>https://www.thermofisher.com/antibody/product/GFP-Antibody-Polyclonal/A-6455<br>https://www.sigmaaldrich.com/JP/en/product/sigma/a5441<br>https://www.abcam.com/products/primary-antibodies/hnrnp-k-antibody-3c2-ab39975.html

Eukaryotic cell lines

Policy information about cell lines and Sex and Gender in Research

Cell line source(s)	Human embryonic kidney (HEK293T/17) cells from ATCC (CRL-11268)<br>https://www.atcc.org/products/crl-11268
Authentication	The cells were purchased from ATCC with material transfer agreement.
Mycoplasma contamination	Cell lines used were not contaminated by Mycoplasma
Commonly misidentified lines<br>(See ICLAC register)	None
