# Supplementary material for: Decryption of sequence, structure, and functional features of SINE repeat elements in SINEUP non-coding RNA-mediated post-transcriptional gene regulation
Source: Nat Commun. 2024 Feb 21;15:1400. doi: 10.1038/s41467-024-45517-3 (PMC10881587; doi:10.1038/s41467-024-45517-3)
Supplement: Supplementary file 2 — Peer Review File [file 41467_2024_45517_MOESM2_ESM.pdf]

nature portfolio

Peer Review File

Decryption of sequence, structure, and functional features of SINE repeat elements in SINEUP non-coding RNA–mediated post-transcriptional gene regulation

Open Access This file is licensed under a Creative Commons Attribution 4.0 International License, which permits use, sharing, adaptation, distribution and reproduction in any medium or format, as long as you give appropriate credit to the original author(s) and the source, provide a link to the Creative Commons license, and indicate if changes were made. In the cases where the authors are anonymous, such as is the case for the reports of anonymous peer reviewers, author attribution should be to 'Anonymous Referee' followed by a clear attribution to the source work. The images or other third party material in this file are included in the article's Creative Commons license, unless indicated otherwise in a credit line to the material. If material is not included in the article's Creative Commons license and your intended use is not permitted by statutory regulation or exceeds the permitted use, you will need to obtain permission directly from the copyright holder. To view a copy of this license, visit http://creativecommons.org/licenses/by/4.0/.

Editorial Note: This manuscript has been previously reviewed at another journal that is not operating a transparent peer review scheme. This document only contains reviewer comments and rebuttal letters for versions considered at *Nature Communications*.

REVIEWERS' COMMENTS

Reviewer #1 (Remarks to the Author):

In the revised version of this manuscript the authors have addressed my comments and clarified my questions.

Reviewer #2 (Remarks to the Author):

In the revised manuscript authors addressed my previous comments and extensively clarified points that were misleading. This results in a much clearer picture and provides convincing evidence for their statements. Additionally, changes to the text and figures make it much more comprehensible.
